# Symmetric Decomposition of Asymmetric Games

**DOI:** 10.1038/s41598-018-19194-4

**Published:** 2018-01-17

**Authors:** Karl Tuyls, Julien Pérolat, Marc Lanctot, Georg Ostrovski, Rahul Savani, Joel Z Leibo, Toby Ord, Thore Graepel, Shane Legg

**Affiliations:** 1 Google DeepMind, 6 Pancras Square, N1C 4AG London, UK; 2 Dept. of Computer Science, University of Liverpool, Ashton Street, L69 3BX Liverpool, UK; 3 Faculty of Philosophy, Oxford University, Woodstock Road, OX2 6GG Oxford, UK

## Abstract

We introduce new theoretical insights into two-population asymmetric games allowing for an elegant symmetric decomposition into two single population symmetric games. Specifically, we show how an asymmetric bimatrix game (*A*,*B*) can be decomposed into its symmetric counterparts by envisioning and investigating the payoff tables (*A* and *B*) that constitute the asymmetric game, as two independent, single population, symmetric games. We reveal several surprising formal relationships between an asymmetric two-population game and its symmetric single population counterparts, which facilitate a convenient analysis of the original asymmetric game due to the dimensionality reduction of the decomposition. The main finding reveals that if *(x,y)* is a Nash equilibrium of an asymmetric game (*A*,*B*), this implies that *y* is a Nash equilibrium of the symmetric counterpart game determined by payoff table *A*, and *x* is a Nash equilibrium of the symmetric counterpart game determined by payoff table *B*. Also the reverse holds and combinations of Nash equilibria of the counterpart games form Nash equilibria of the asymmetric game. We illustrate how these formal relationships aid in identifying and analysing the Nash structure of asymmetric games, by examining the evolutionary dynamics of the simpler counterpart games in several canonical examples.

## Introduction

We are interested in analysing the Nash structure and evolutionary dynamics of strategic interactions in multi-agent systems. Traditionally, such interactions have been studied using single population replicator dynamics models, which are limited to symmetric situations, i.e., players have access to the same set of strategies and the payoff structure is symmetric as well^1^. For instance, Walsh *et al*. introduce an empirical game theory methodology (also referred to as heuristic payoff table method) that allows for analysing multiagent interactions in complex multiagent games^2,3^. This method has been extended by others and been applied e.g. in continuous double auctions, variants of poker and multi-robot systems^1,4–9^. Similar evolutionary methods have been applied to the modelling of human cooperation, language, and complex social dilemma’s^10–16^. Though these evolutionary methods have been very useful in providing insights into the type and form of interactions in such systems, the underlying Nash structure, and evolutionary dynamics, the analysis is limited to symmetric situations, i.e., players or agents can be interchanged and have access to the same strategy set, in other words there are no different roles for the various agents involved in the interactions (e.g. a seller vs a buyer in an auction). As such this method is not directly applicable to asymmetric situations in which the players can choose strategies from different sets of actions, with asymmetric payoff structures. Many interesting multiagent scenarios involve asymmetric interactions though, examples include simple games from game theory such as e.g. the Ultimatum Game or the Battle of the Sexes and more complex board games that can involve various roles such as Scotland Yard, but also trading on the internet for instance can be considered asymmetric.

There exist approaches that deal with asymmetry in multiagent interactions, but they usually propose to transform the asymmetric game into a symmetric game, with new strategy sets and payoff structure, which then can be analysed again in the context of symmetric games. This is indeed a feasible approach, but not easily scalable to the complex interactions mentioned before, nor is it practical or intuitive to construct a new symmetric game before the asymmetric one can be analysed in full. The approach we take in this paper does not require constructing a new game and is theoretically underpinned, revealing some new interesting insights in the relation between the Nash structure of symmetric and asymmetric games.

Analysing multiagent interactions using evolutionary dynamics, or replicator dynamics, provides not only valuable insights into the (Nash) equilibria and their stability properties, but also sheds light on the behaviour trajectories of the involved agents and the basins of attraction of the equilibrium landscape^1,4,15,17,18^. As such it can be a very useful tool to analyse the Nash structure and dynamics of several interacting agents in a multiagent system. However, when dealing with asymmetric games the analysis quickly becomes tedious, as in this case we have a coupled system of replicator equations, and changes in the behaviour of one agent immediately change the dynamics in the linked replicator equation describing the behaviour of the other agent, and vice versa. This paper sheds new light on asymmetric games, and reveals a number of theorems, previously unknown, that allow for a more elegant analysis of asymmetric multiagent games. The major innovation is that we decouple asymmetric games in their *symmetric counterparts*, which can be studied in a symmetric fashion using symmetric replicator dynamics. The Nash equilibria of these symmetric counterparts are formally related to the Nash equilibria of the original asymmetric game, and as such provide us with a means to analyse the asymmetric game using its symmetric counterparts. Note that we do not consider asymmetric replicator dynamics in which both intra-species (within a population) and inter-species interactions (between different populations) take place^19^, but we only consider inter-species interactions in which two different roles interact, i.e., truly asymmetric games^20^.

One of our main findings is that the *x strategies* (player 1) and the *y strategies* (player 2) of a mixed Nash equilibrium of full support in the original asymmetric game, also constitute Nash equilibria in the symmetric counterpart games. The symmetric counterpart of player 1 (*x*) is defined on the payoff of player 2 and vice versa. We prove that for full support strategies, Nash equilibria of the asymmetric game are pairwise combinations of Nash equilibria of the two symmetric counterparts. Then, we show that this property stands without the assumption of full support as well. Though this analysis does not allow us to visualise the evolutionary dynamics of the asymmetric game itself, it does allow us to identify its Nash equilibria by investigating the evolutionary dynamics of the counterparts. As such we can easily distinguish Nash equilibria from other restpoints in the asymmetric game and get an understanding of its underlying Nash structure.

The paper is structured as follows: we first describe related work, then we continue with introducing essential game theoretic concepts. Subsequently, we present the main contributions and we illustrate the strengths of the theory by carrying out an evolutionary analysis on four canonical examples. Finally, we discuss the implications and provide a deeper understanding of the theoretical results.

## Related Work

The most straightforward and classical approach to asymmetric games is to treat agents as evolving separately: one population per player, where each agent in a population interacts by playing against agent(s) from the other population(s), i.e. co-evolution^21^. This assumes that players of these games are always fundamentally attached to one role and never need to know/understand how to play as the other player. In many cases, though, a player may want to know how to play as either player. For example, a good chess player should know how to play as white or black. This reasoning inspired the role-based symmetrization of asymmetric games^22^.

The role-based symmetrization of an arbitrary bimatrix game defines a new (extensive-form) game where before choosing actions the role of the two players are decided by uniform random chance. If two roles are available, an agent is assigned one specific role with probability $\frac{1}{2}$. Then, the agent plays the game under that role and collects the role-specific payoff appropriately. A new strategy space is defined, which is the product of both players’ strategy spaces, and a new payoff matrix computing (expected) payoffs for each combination of pure strategies that could arise under the different roles. There are relationships between the sets of evolutionarily stable strategies and rest points of the replicator dynamics between the original and symmetrized game^19,23^.

This single-population model forces the players to be general: able to devise a strategy for each role, which may unnecessarily complicate algorithms that compute strategies for such players. In general, the payoff matrix in the resulting role-based symmetrization is *n*! (*n* being the number of agents) times larger due to the number of permutations of player role assignments. There are two-population variants that formulate the problem slightly differently: a new matrix that encapsulates both players’ utilities assigns 0 utility to combinations of roles that are not in one-to-one correspondence with players^24^. This too, however, results in an unnecessarily larger (albeit sparse) matrix.

Lastly, there are approaches that have structured asymmetry, that arises due to ecological constraints such as locality in a network and genotype/genetic relationships between population members^25^. Similarly here, replicator dynamics and their properties are derived by transforming the payoff matrix into a larger symmetric matrix.

Our primary motivation is to enable analysis techniques for asymmetric games. However, we do this by introducing new *symmetric counterpart dynamics* rather than using standard dynamics on a symmetrised game. Therefore, the traditional role interpretation as well as any method that enlarges the game for the purpose of obtaining symmetry is unnecessarily complex for our purposes. Consequently, we consider the original co-evolutionary interpretation, and derive new (lower-dimensional) strategy space mappings.

## Preliminaries and Methods

In this section we concisely outline (evolutionary) game theoretic concepts necessary to understand the remainder of the paper^23,26,27^. We briefly specify definitions of Normal Form Games and solution concepts such as Nash Equilibrium in a single population game and in a two-population game. Furthermore, we introduce the Replicator Dynamics (RD) equations for single and two population games and briefly discuss the concept of Evolutionary Stable Strategies (ESS) introduced by Smith and Price in 1973^28–30^.

### Normal Form Games and Nash Equilibrium

**Definition**. *A two-player Normal Form Game* (*NFG*) *G is a 4*-*tuple G* = (*S*_1_, *S*_2_, *A*, *B*), *with pure strategy sets S*_1_ *and S*_2_ *for player 1*, *respectively player 2*, *and corresponding payoff tables A and B*. *Both players choose their pure strategies* (*also called actions*) *simultaneously*.

The payoffs for both players are represented by a bimatrix (*A*, *B*), which gives the payoff for the row player in *A*, and the column player in *B* (see Table 1 for a two strategy example). Specifically, the row player chooses one of the two rows, the column player chooses one of the columns, and the outcome of their joint strategy determines the payoff to both.

**Table 1:** General payoff bimatrix (A, B) for a two-player two-action normal form game, where player 1 can choose between actions *A*_1_ and *A*_2_, and player 2 can choose between actions *B*_1_ and *B*_2_.

		Player 2	
		*B*_*1*_	*B*_*2*_
Player 1	*A*_*1*_	*a*_11_, *b*_11_	*a*_12_, *b*_12_
	*A*_*2*_	*b*_21,_ *a*_21_	*a*_22_, *b*_22_

In case *S*_1_ = *S*_2_ and *A* = *B*^*T*^ the players are interchangeable and we call the game symmetric. In case at least one of these conditions is not met we have an asymmetric game. In classical game theory the players are considered to be individually rational, in the sense that each player is perfectly logical trying to maximise their own payoff, assuming the others are doing likewise. Under this assumption, the Nash equilibrium (NE) solution concept can be used to study what players will reasonably choose to do.

We denote a strategy profile of the two players by the tuple (*x*, *y*) ∈ Δ*S*_1_ × Δ*S*_2_, where Δ*S*_1_, Δ*S*_2_ are the sets of mixed strategies, that is, distributions over the pure strategy sets or action sets. The strategy *x* (respectively *y*) is represented as a vector in ${{\mathbb{R}}}^{|{S}_{1}|}$ (respectively ${{\mathbb{R}}}^{|{S}_{2}|}$) where each entry is the probability of playing the corresponding action. The payoff associated with player 1 is *x*^*T*^*Ay* and *x*^*T*^*By* is the payoff associated with player 2. A strategy profile (*x*,*y*) now forms a NE if no single player can do better by unilaterally switching to a different strategy. In other words, each strategy in a NE is a best response against all other strategies in that equilibrium. Formally we have,

**Definition**. *A strategy profile* (*x*,*y*) *is a Nash equilibrium*, *iff the following holds*:

$$\forall x^{\prime} \,\in \,\Delta {S}_{1},\,{x}^{T}Ay\ge {x^{\prime} }^{T}Ay\,and\,\forall y^{\prime} \,\in \,\Delta {S}_{2},\,{x}^{T}\,By\ge {x}^{T}By^{\prime} $$

In the following, we will write *NE*(*A*, *B*) for the set of Nash equilibria of the game *G* = (*S*_1_, *S*_2_, *A*, *B*). Furthermore, a Nash equilibrium is said to be pure if only one strategy of the strategy set is played and we will say that it is completely mixed if all pure strategies are played with a non-zero probability.

In evolutionary game theory, games are often considered with a single population. In other words, a player is playing against itself and only a single payoff table *A* is necessary to define the game (note that this definition only makes sense when |*S*_1_| = |*S*_2_| = *n*). In this case, the payoff received by the player is *x*^*T*^*Ax* and the following definition describes the Nash equilibrium:

**Definition**. *In a single population game*, *a strategy x is a Nash equilibrium*, *iff the following holds*:

$$\forall x^{\prime} ,{x}^{T}Ax\ge {x^{\prime} }^{T}Ax$$

In this single population case, we will write that *x *∈ *NE*(*A*).

### Replicator Dynamics

Replicator Dynamics in essence are a system of differential equations that describe how a population of pure strategies, or replicators, evolve through time^26,32^. In their most basic form they correspond to the biological *selection* principle, i.e. survival of the fittest. More specifically the *selection* replicator dynamic mechanism is expressed as follows:

1 $$\frac{d{x}_{i}}{dt}={x}_{i}[(Ax{)}_{i}-{x}^{T}Ax]$$

Each replicator represents one (pure) strategy *i*. This strategy is inherited by all the offspring of the replicator. *x*_*i*_ represents the density of strategy *i* in the population, *A* is the payoff matrix which describes the different payoff values each individual replicator receives when interacting with other replicators in the population. The state of the population *x* can be described as a probability vector *x* = (*x*_1_, *x*_2_, ..., *x*_*n*_) which expresses the different densities of all the different types of replicators in the population. Hence (*Ax*)_*i*_ is the payoff which replicator *i* receives in a population with state *x* and *x*^*T*^*Ax* describes the average payoff in the population. The support *I*_*x*_ of a strategy is the set of actions (or pure strategies) that are played with a non-zero probability *I*_*x*_ = {*i* |*x*_*i*_ > 0}.

In essence this equation compares the payoff a strategy receives with the average payoff of the entire population. If the strategy scores better than average it will be able to replicate *offspring*, if it scores lower than average its presence in the population will diminish and potentially approach extinction. The population remains in the simplex (∑_*i*_*x*_*i*_ = 1) since ∑_*i*_(*dx*_*i*_)/(*dt*) = 0.

### Evolutionary Stable Strategies

Originally, an Evolutionary Stable Strategy was introduced in the context of a symmetric single population game^28,32^ (as introduced in the previous section), though this can be extended to multi-population games as well as defined in the next section^23,33^. Imagine a population of simple agents playing the same strategy. Assume that this population is invaded by a different strategy, which is initially played by a small proportion of the total population. If the reproductive success of the new strategy is smaller than the original one, it will not overrule the original strategy and will eventually disappear. In this case we say that the strategy is *evolutionary stable* (ESS) against this newly appearing strategy. In general, we say a strategy is ESS if it is robust against evolutionary pressure from any appearing mutant replicator not yet present in the population (or only with a very small fraction).

### Asymmetric Replicator Dynamics

We have assumed replicators come from a single population, which makes the model only applicable to symmetric games. One can now wonder how the previous introduced equations extend to asymmetric games. Symmetry assumes that strategy sets and corresponding payoffs are the same for all players in the interaction. An example of an asymmetric game is the famous Battle of the Sexes (BoS) game illustrated in Table 2. In this game both players do have the same strategy set, i.e., go to the opera or go to the movies, however, the corresponding payoffs for each are different, expressing the difference in preferences that both players have in their respective roles.

**Table 2:** Payoff bimatrix for the Battle of the Sexes game. Strategies *O* and *M* correspond to going to the Opera and going to the Movies respectively.

	*O*	*M*
*O*	3, 2	0, 0
*M*	0, 0	2, 3

If we would like to carry out a similar evolutionary analysis as before we will now need two populations, one for each player over its respective strategy set, and we need to use the asymmetric or coupled version of the replicator dynamics, i.e.,

**Definition**.

2 $$\frac{d{x}_{i}}{dt}={x}_{i}[(Ay{)}_{i}-{x}^{T}Ay]\quad \quad {and}\quad \quad \frac{d{y}_{i}}{dt}={y}_{i}[({x}^{T}B{)}_{i}-{x}^{T}By]$$

with payoff tables *A* and *B*, respectively for player 1 and 2. In case *A* = *B*^*T*^ the equations reduce to the single population model.

### Symmetric Counterpart Replicator Dynamics

We now introduce a new concept, the *symmetric counterpart* replicator dynamics (SCRD) of asymmetric replicator equations. We consider the two payoff tables *A* and *B* as two independent games that are no longer coupled, and in which both players participate. In the first counterpart game all players choose their strategy according to distribution *y*, the original strategy or replicator distribution for the 2nd population, or player 2, and in the second counterpart game all players choose their strategy according to distribution *x*, the original strategy or replicator distribution for the 1st population, or player 1. This gives us the following two sets of replicator equations:

3 $$\frac{d{y}_{i}}{dt}={y}_{i}[(Ay{)}_{i}-{y}^{T}Ay]$$

and

4 $$\frac{d{x}_{i}}{dt}={x}_{i}[({x}^{T}B{)}_{i}-{x}^{T}Bx]$$

In the results Section we will introduce some remarkable relationships between the equilibria of asymmetric replicator equations and the equilibria of their symmetric counterpart equations, which facilitates, and substantially simplifies, the analysis of the Nash structure of asymmetric games.

### Visualising evolutionary dynamics

One can visualise the replicator dynamics in a directional field and trajectory plot, which provides useful information about the equilibria, flow of dynamics and basins of attraction. As long as we stay in the realm of 2-player 2-action games this can be achieved relatively easily by plotting the probability with which player 1 plays its first action on the x-axis, and the probability with which player 2 plays its first action on the y-axis. Since there are only 2 actions for each player, this immediately gives a complete image of the dynamics over all strategies, since the probability for the second action *a*_2_ to be chosen is one minus the first. By means of example we show a directional field plot here for the famous Prisoner’s dilemma game (game illustrated in Table 3).

**Table 3:** Payoff matrix for the Prisoner’s Dilemma game. Strategies *D* and *C* correspond to the actions *Defect* and *Cooperate*.

	*C*	*D*
*C*	3, 3	0, 5
*D*	5, 0	1, 1

**Figure 1:**
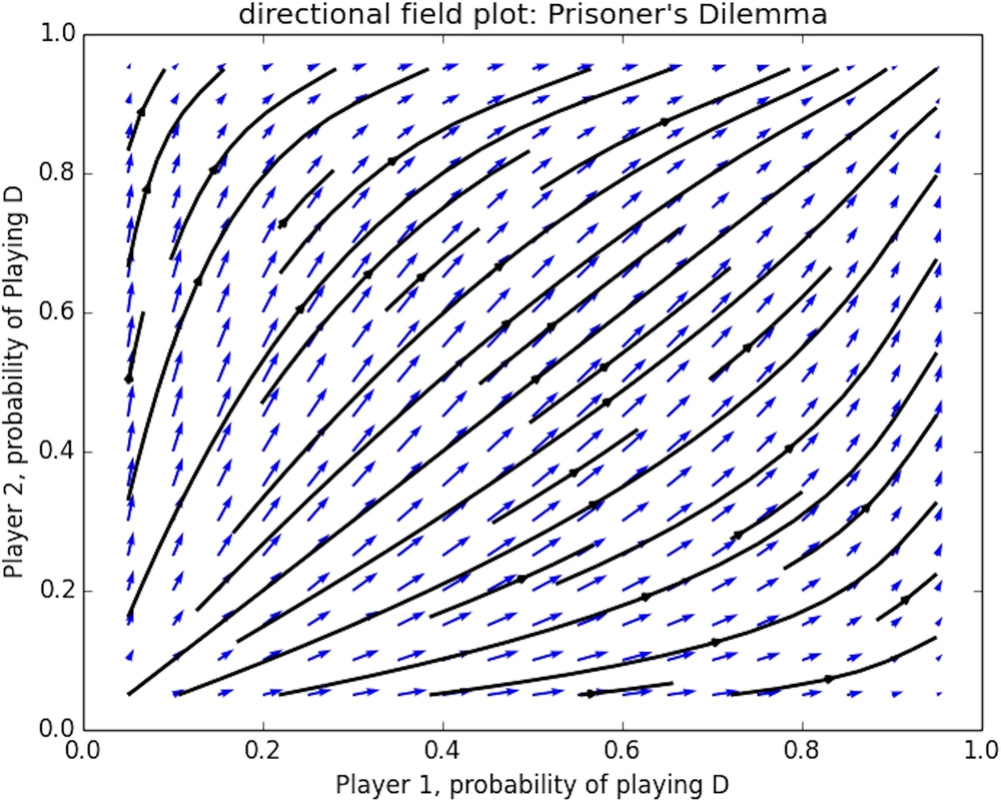
Directional field plot of the Prisoner’s Dilemma game.

The directional field plot, and corresponding trajectories, are shown in Fig. 1. For both players the axis represents the probability with which they play *Defect* (D). As can be observed all dynamics are absorbed by the pure Nash equilibrium (*D*, *D*) in which both players defect.

**Figure 2:**
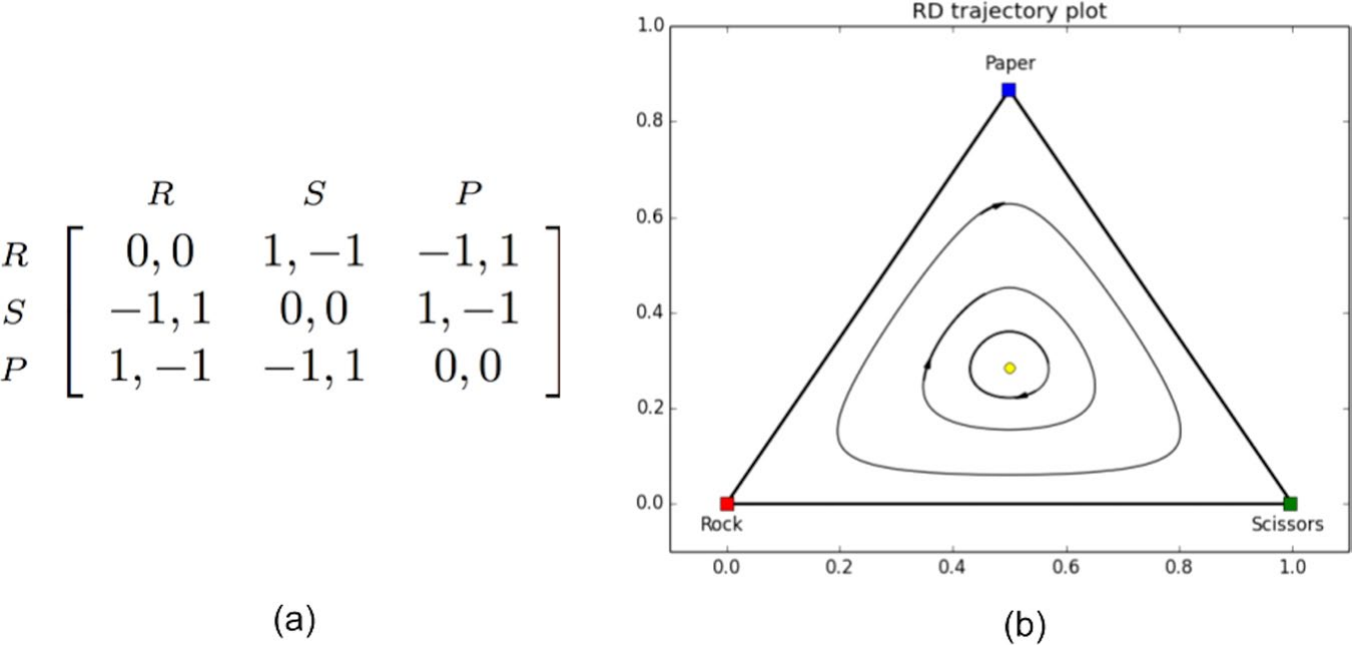
(**a**) Payoff matrix for the Rock-Paper-Scissors game. Strategies *R*, *S* and *P* correspond to playing respectively *R*ock, *S*cissors, *P*aper. (**b**) Σ_3_ Trajectory plot of the Rock-Paper-Scissors game. The Nash equilibrium is marked with a full yellow dot.

Unfortunately, we cannot use the same type of plot illustrating the dynamics when we consider more than two strategies. However, if we move to single population games we can easily rely on a simplex plot. In the case of a two population game the situation become tedious as we will discuss later. Specifically, the set of probability distributions over *n* elements can be represented by the set of vectors (*x*_1_, ..., *x*_*n*_) $\in \,{{\mathbb{R}}}^{n}$, satisfying *x*_1_, ..., *x*_*n*_ ≥ 0 and ∑_*i*_*x*_*i*_ = 1. This can be seen to correspond to an *n* − 1-dimensional structure called a simplex Σ_*n*_ (or simply Σ, when *n* is clear from the context). In many of the figures throughout the paper we use Σ_3_, projected as an equilateral triangle. For example, consider the single population *Rock-Paper-Scissors* game, described by the payoff matrix shown in Fig. 2a.

The game has one completely mixed Nash equilibrium, being $(\frac{1}{3},\frac{1}{3},\frac{1}{3})$. In Fig. 2b we have plotted the replicator equations Σ_3_ trajectory plot for this game. Each of the corners of the simplex corresponds to one of the pure strategies, i.e., {*Rock*, *Paper*, *Scissors*}. For three strategies in the strategy simplex we then plot a trajectory illustrating the flow of the replicator dynamics. As can be observed from the plot, trajectories of the dynamics cycle around the mixed Nash equilibrium, which is not ESS and not asymptotically stable.

**Figure 3:**
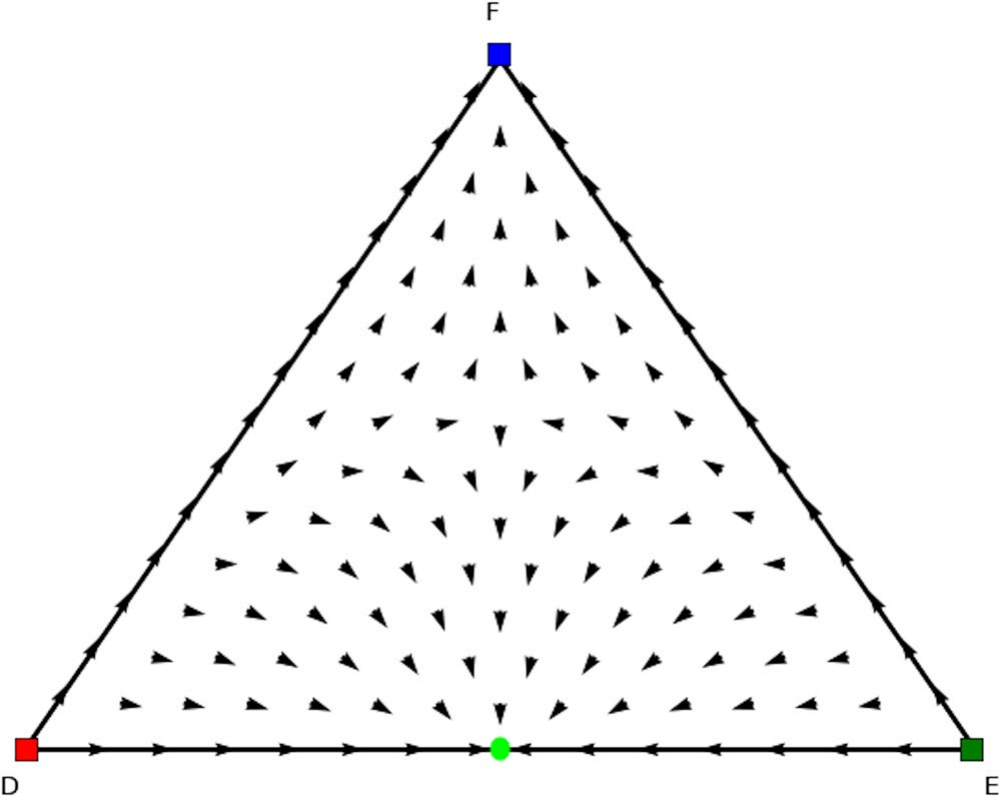
ESS.

**Figure 4:**
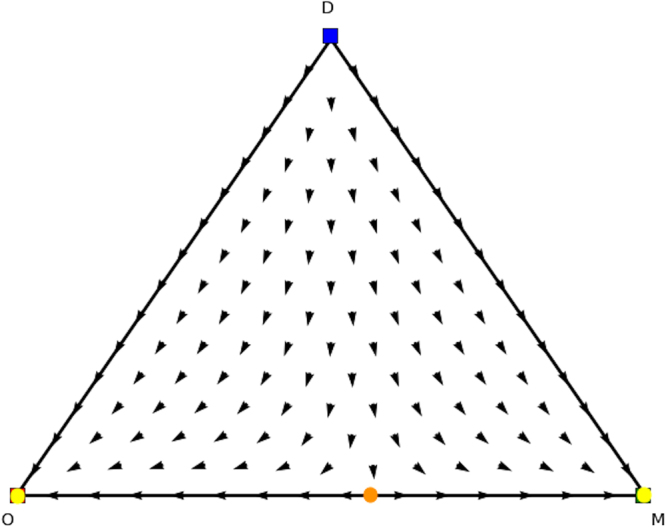
NE but not ESS.

**Figure 5:**
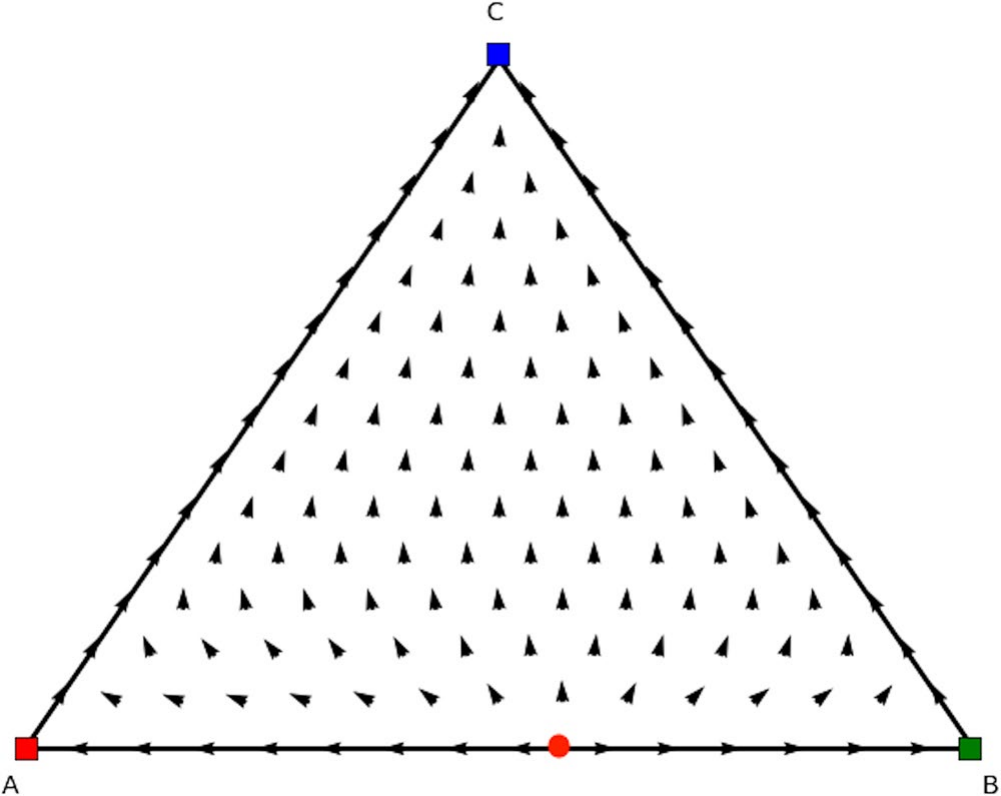
Rest point but not NE.

In fact, three categories of rest points can be discerned in single population replicator dynamics (see Figs 3, 4 and 5). Figure 3 displays a stable Nash equilibrium called an Evolutionary Stable Strategy (ESS). An ESS is an attractor of the RD dynamical system defined in the previous section and has been one of the main foci of evolutionary game theory. The second type of rest points are the ones that are Nash but not ESS (Fig. 4). These rest points are not an attractor of the RD but they have a specific form. Specifically, if a strategy is a Nash equilibrium, all the actions that are not part of the support are dominated, i.e., the support is invariant under the RD, which means that the fraction of a strategy cannot become non-zero if it is zero at some point. The third category that can occur is illustrated in Fig. 5. Those rest points are not Nash and thus there is an action outside of the support that is dominant. Thus, the flow will leave from points in the close vicinity of the rest point, which is called a *source*.

## Results

In the following, we first present our main findings, formally relating Nash equilibria in asymmetric 2-player games with the Nash equilibria that can be found in the corresponding counterpart games. We also examine the stability properties of the corresponding rest points of the replicator dynamics in these games. Then we experimentally illustrate these findings in some canonical examples.

### Theoretical Findings

In this section, we prove the following result: if (*x*, *y*) ∈ *NE*(*A*, *B*) (where *x* and *y* have the same support), then *x *∈ *NE*(*B*^Τ^) and *y *∈ *NE*(*A*). In addition, we prove that the reverse is true: if *x *∈ *NE*(*B*^Τ^) and *y *∈ *NE*(*A*) (where *x* and *y* have the same support) then (*x*,*y*) ∈ *NE*(*A*,*B*). We will prove this result in two steps (Theorem 1 and its generalization Theorem 2).

The theorems introduced apply to games where both players can play the same number of actions (i.e. square games). This condition can be weakened by adding dominated strategies to the player having the smallest number of actions (see the extended Battle of the Sexes example in the experimental section). Thus, without loss of generality, the theory will focus on square games. To begin, we state an important well-known property of Nash equilibria, that has been given different names; Gintis calls it fundamental theorem of Nash equilibria^32^. For sake of completeness, we provide a proof.

#### **Property 1.**

*Let the strategy profile* (*x*, *y*) *be a Nash equilibrium of an asymmetric normal form game* (*A*, *B*), *and denote I*_*z*_ = {*i* | *z*_*i*_ > 0} *the support of a strategy z*. *Then*,

5 $${z}^{{\rm{{\rm T}}}}Ay={x}^{{\rm{{\rm T}}}}Ay\,for\,all\,z\,such\,that\,{I}_{z}\subset {I}_{x},\,\,and$$

6 $${x}^{{\rm{{\rm T}}}}Bz={x}^{{\rm{{\rm T}}}}By\,for\,all\,z\,such\,that\,{I}_{z}\subset {I}_{y}\mathrm{.}$$

#### *Proof*.

This result is widely known. We provide it as it is a basis of our theoretical results and for the sake of completeness.

If *x* and *y* constitute a Nash equilibrium then, by definition *z*^Τ^*Ay* ≤ *x*^Τ^*Ay*,∀*z*. Let us suppose that there exists a *z* with *I*_*z*_ ⊂ *I*_*x*_ such that *z*^Τ^*Ay* < *x*^Τ^*Ay*. Then there is a *i* ∈ *I*_*z*_ ⊂ *I*_*x*_ satisfying (*Ay*)_*i*_ < *x*^Τ^*Ay*, and we get ${x}^{{\rm{{\rm T}}}}Ay=\sum _{i\in {I}_{x}}{x}_{i}{(Ay)}_{i} < \sum _{i\in {I}_{x}}{x}_{i}{x}^{{\rm{{\rm T}}}}Ay={x}^{{\rm{{\rm T}}}}Ay$, which is a contradiction, proving the first claim. The claim for *B* follows analogously.◽

#### **Property 2**.

*Let the strategy x be a Nash equilibrium of a single population game A*. *Then*,

7 $${z}^{{\rm T}}Ax={x}^{{\rm T}}Ax\,for\,all\,z\,such\,that\,{I}_{z}\subset {I}_{x}\mathrm{.}$$

#### *Proof*.

The proof is similar to the proof of Property 1.◽

This property will be useful in the steps of the proofs that follow. We now present our first main result: a correspondence between the Nash equilibria of full support in the asymmetric game with those of full support in the counterpart games. Theorem 2 subsumes this result and we introduce this simpler version first for the sake of readability.

#### ***Theorem 1.***

*If strategies x and y constitute a Nash equilibrium of an asymmetric normal form game G =* (*S*_*1*_, *S*_*2*_, *A*, *B*), *with both x*_*i*_ *> 0 and y*_*j*_ *> 0 for all i*, *j* (*full support*), *and |S*_*1*_*| = |S*_*2*_*| = n*, *then it holds that x is a Nash equilibrium of the single population game B*^*T*^ *and y is a Nash equilibrium of the single population game A*. *The reverse is also true*.

#### *Proof* .

This result follows naturally from Property 1 and is implied by Theorem 2.

We start by assuming that *x* and *y* constitute a full support Nash equilibrium of the asymmetric game (*A*, *B*). By Property 1 and since *x* and *y* have full support, we know that:

$$Ay={\mathrm{(1,}\mathrm{...,}\mathrm{1)}}^{T}\,\mathop{max}\limits_{i\in \mathrm{\{1,...,}n\}}\,{(Ay)}_{i}\quad \mathrm{and},\quad {x}^{T}B=\mathrm{(1,}\,\mathrm{...,}\,\mathrm{1)}\,\mathop{max}\limits_{i\in \mathrm{\{1,...,}n\}}\,{({x}^{T}B)}_{i}$$

From this we also know that *y*^*T*^*Ay* = (*Ay*)_*i*_ (since the (*Ay*)_*i*_ are equal for all *I*’s in the vector *Ay*, so multiplying *Ay* with *y*^*T*^ will yield the same number ${{\rm{\max }}}_{i}\,{(Ay)}_{i}$), and similarly (*x*^*T*^*B*)_*i*_ = *x*^*T*^*Bx* (and thus (*B*^*T*^*x*)_*i*_ = *x*^*T*^*B*^*T*^*x*), implying that:

$$\forall y^{\prime} ,{y}^{T}Ay={y^{\prime} }^{T}Ay\quad \mathrm{and},\quad \forall x^{\prime} ,{x}^{T}{B}^{T}x={x^{\prime} }^{T}{B}^{T}x$$

This concludes the proof.◽

For the first counterpart game this means that the players will use the *y* part of the Nash equilibrium of player 2 of the original asymmetric game, in the symmetric counterpart game determined by payoff table *A*. And similarly, for the second counterpart game this means that players will play according to the *x* part of the Nash equilibrium of player 1 of the original asymmetric game, in the symmetric game determined by payoff table *B*. As such both players consider a symmetric version of the asymmetric game, for which this *y* component and *x* component constitute a Nash equilibrium in the two new respective symmetric games.

In essence, these two symmetric counterpart games can be considered as a decomposition of the original asymmetric game, which gives us a means to illustrate in a smaller strategy space where the mixed and pure equilibria are located.

A direct consequence of Theorem 1 is the following corollary that gives insights on the geometrical structure of Nash equilibrium,

#### **Corollary 1**.

*Combinations of Nash equilibria of full support of the games corresponding to the symmetrical counterparts of the original asymmetric game also form Nash equilibria of full support in this asymmetric game*.

#### *Proof*.

This is a direct consequence of Theorem 1.◽

The next theorem explores the case where the equilibrium is not of full support. We prove that the theorem stands if the strategies of both players have the same support. Indeed, the first theorem requires that both players play all actions with a positive probability, here we will only require that they play the actions with the same index with a positive probability. We say that *x* and *y* have the same support if the set of played actions *I*_*x*_ = {*i* | *x*_*i*_ > 0} and *I*_*y*_ = {*i* | *y*_*i*_ > 0} are equal.

#### **Theorem 2**.

*Strategies x and y constitute a Nash equilibrium of an asymmetric game G =* (*S*_*1*_, *S*_*2*_, *A*, *B*) *with the same support* (*i*.*e*. *I*_*x*_ *= I*_*y*_) *if and only if x is a Nash equilibrium of the single population game B*^*T*^, *y is a Nash equilibrium of the single population game A and I*_*x*_ *= I*_*y*_.

#### *Proof*.

We start by assuming that *x* and *y* constitute a Nash equilibrium of same support (*I*_*x*_ = *I*_*y*_) of the asymmetric game (*A*, *B*). By Property 1, and since *x* and *y* have the same support, we know that:

8 $${z}^{{\rm{{\rm T}}}}Ay={x}^{{\rm{{\rm T}}}}Ay\,{\rm{for}}\,{\rm{all}}\,z\,{\rm{such}}\,{\rm{that}}\,{I}_{z}\subset {I}_{x},\,{\rm{and}}$$

9 $${x}^{{\rm{{\rm T}}}}Bz^{\prime} ={x}^{{\rm{{\rm T}}}}By\,{\rm{for}}\,{\rm{all}}\,z^{\prime} \,{\rm{such}}\,{\rm{that}}\,{I}_{z^{\prime} }\subset {I}_{y}\mathrm{.}$$

Implying that *y*^Τ^*Ay* = *x*^Τ^*Ay* and *x*^Τ^*Bx* = *x*^Τ^*By* (by setting *z* = *y* and *z*′ = *x*). Then, from the Nash equilibrium condition we can write:

$$\forall x^{\prime} \,\in \,\Delta {S}_{1},\,{x}^{T}Ay\ge {x^{\prime} }^{T}Ay\,{\rm{and}}\,\forall y^{\prime} \,\in \,\Delta {S}_{2},\,{x}^{T}By\ge {x}^{T}By^{\prime} $$

$${\rm{\forall }}y{\rm{^{\prime} }}\,\in \,{\rm{\Delta }}{S}_{2},\,{y}^{{\rm{T}}}Ay\ge {y{\rm{^{\prime} }}}^{{\rm{T}}}Ay\,{\rm{a}}{\rm{n}}{\rm{d}}\,{\rm{\forall }}x{\rm{^{\prime} }}\,\in \,{\rm{\Delta }}{S}_{1},\,{x}^{{\rm{T}}}Bx\ge {x}^{T}Bx{\rm{^{\prime} }}$$

$$\forall y^{\prime} \,\in \,\Delta {S}_{2},\,{y}^{{\rm{T}}}Ay\ge {y^{\prime} }^{T}Ay\,{\rm{and}}\,\forall x^{\prime} \,\in \,\Delta {S}_{1},\,{x}^{{\rm{T}}}{B}^{{\rm{T}}}x\ge {x^{\prime} }^{T}{B}^{{\rm{T}}^{\prime} }$$

which implies that *y* is a Nash equilibrium of *B*^Τ^ and *x* is a Nash equilibrium of *A*.

The proof of the other direction follows similar mechanics and uses Property 2. Let us now assume that *y* is a Nash equilibrium of *B*^Τ^ and *x* is a Nash equilibrium of *A* with *I*_*x*_ = *I*_*y*_. Then, from Property 2 we have:

10 $${z}^{{\rm{{\rm T}}}}Ay={y}^{{\rm{{\rm T}}}}Ay\,{\rm{for}}\,{\rm{all}}\,z\,{\rm{such}}\,{\rm{that}}\,{I}_{z}\subset {I}_{y},\,{\rm{and}}$$

11 $${z^{\prime} }^{{\rm T}}{B}^{{\rm T}}x={x}^{{\rm T}}{B}^{{\rm T}}x\,{\rm{for}}\,{\rm{all}}\,z^{\prime} \,{\rm{such}}\,{\rm{that}}\,{I}_{z^{\prime} }\subset {I}_{x}\mathrm{.}$$

In particular we get *y*^Τ^*Ay* = *x*^Τ^*Ay* and *x*^Τ^*Bx* = *x*^Τ^*By* (by setting *z* = *x* and *z*′ = *y*). From the Nash equilibrium condition of the single population games we can write:

$$\forall y^{\prime} \in \Delta {S}_{2},\,{y}^{{\rm{T}}}Ay\ge {y^{\prime} }^{T}Ay\,{\rm{and}}\,\forall x^{\prime} \,\in \,\Delta {S}_{1},\,{x}^{{\rm{T}}}{B}^{{\rm{T}}}x\ge {x^{\prime} }^{{\rm{T}}}{B}^{{\rm{T}}}x$$

$$\forall y^{\prime} \in \Delta {S}_{2},\,{y}^{{\rm{T}}}Ay\ge {y^{\prime} }^{T}Ay\,{\rm{and}}\,\forall x^{\prime} \in \Delta {S}_{1},{x}^{{\rm{T}}}Bx\ge {x}^{T}Bx^{\prime} $$

$$\forall x^{\prime} \in \Delta {S}_{1},{x}^{T}Ay\ge {x^{\prime} }^{T}Ay\,{\rm{and}}\,\forall y^{\prime} \in \Delta {S}_{2},\,{x}^{T}By\ge {x}^{T}By^{\prime} $$

which concludes the proof.◽

#### ***Corollary 2***.

*Strategies x and y constitute a pure* (*strict*) *Nash equilibrium of an asymmetric normal form game G =* (*S*_*1*_, *S*_*2*_, *A*, *B*), *with support on the strategy with the same index in their respective strategy sets S*_*1*_ *and S*_*2*_, *if and only if*, *y and x are also pure* (*strict*) *Nash equilibria of the counterpart games defined by A*,

12 $$\frac{d{y}_{i}}{dt}={y}_{i}((Ay{)}_{i}-{y}^{T}Ay)=0$$

*and B*,

13 $$\frac{d{x}_{i}}{dt}={x}_{i}(({x}^{T}B{)}_{i}-{x}^{T}Bx)=0$$

#### *Proof*.

This is a direct consequence of Theorem 2.◽

The theorems can only be used for equilibria in the counterpart games with matching supports (*I*_*x*_ = *I*_*y*_) from both players. One can work around this condition though by simply permuting the actions of one player in matrix *A* and *B* to study all configurations of supports of the same cardinality. To be precise, we need to analyze all the counterpart games defined by *A*_Σ_ = *A*Σ and ${B}_{\Sigma }^{T}=(B{\rm{\Sigma }}{)}^{T}$ for all permutation matrices Σ. This technique is sufficient to study non-degenerate games, as in a non-degenerate game all Nash equilibria have a support of same size (in a non-degenerate game if (*x*, *y*) is a Nash equilibrium then |*I*_*x*_| = |*I*_*y*_|^34^).

### Stability Analysis

We can now examine the stability of the pure Nash equilibria discussed in the previously derived theorems.

#### **Corollary 3**.

*Strategy y is a strict Nash equilibrium of the first counterpart game defined by A and strategy x* is a strict Nash equilibrium of the second counterpart game defined by B, if and only if, (*x*, *y*) is a locally asymptotically stable equilibrium and a two-species ESS of the asymmetric normal form game *G* = (*S*_1_, *S*_2_, *A*, *B*) with support on the strategy with the same index in their respective strategy sets *S*_1_ and *S*_2_.

#### *Proof*.

This a direct consequence of Corollary 2. More specifically, from Corollary 2 we know that (*x*, *y*) is a strict Nash equilibrium of *G*. It has been shown that (*x*, *y*) is a strict Nash equilibrium of *G* iff it is a two-species ESS^19,20,27^.◽

## Experimental illustration

We will now illustrate how the theoretical links between asymmetric games and their counterpart symmetric replicator dynamics facilitate analysis of asymmetric multiagent games, and provide a convenient tool to get insight into their equilibrium landscape. We do this for several examples. The first example concerns the Battle of the Sexes game to illustrate the intuition behind the results. The second example extends the Battle of the Sexes game with one strategy for one of the players, illustrating the permutation argument of the theorems and how to apply the results in case of a non-square game. The third example is a bimatrix game generated in the context of a multiagent learning algorithm called PSRO (Policy Space Response Oracles^9^) and concerns Leduc Poker. This algorithm produces normal-form “empirical games” which each correspond to an extensive-form game with a reduced strategy space, using incremental best response learning. Finally, the last asymmetric game illustrates the theorems for a single mixed equilibrium of full support, while its counterpart games have many more equilibria.

A fundamental complexity arises when using the evolutionary dynamics of a 2-player asymmetric game to analyse its equilibrium structure, as the dynamics for the two players is intrinsically coupled and high-dimensional. While one could fix a player’s strategy and consider the induced dynamics for the other player in its respective strategy simplex, a static trajectory plot of this would not faithfully represent the complexity of the full 2-player dynamics. To gain a somewhat more complete intuitive picture, one can represent this dynamics as a movie, showing the change in induced dynamics for one player, as one varies the (fixed) strategy for the other (we will illustrate this in the PSRO-produced game on Leduc Poker).

The theorems introduced in the previous section help to overcome this problem, and allow to analyse the evolutionary dynamics of the symmetric counterpart games instead of the asymmetric game itself, revealing the landscape of Nash equilibria, which seriously simplifies the analysis.

### Battle of the Sexes

Symmetry assumes that strategy sets and corresponding payoffs are the same for all players in the interaction. An example of an asymmetric game is the Battle of the Sexes (BoS) game illustrated in Table 2. In this game both players do have the same strategy set, i.e., go to the *opera* or go to the *movies*, however, the corresponding payoffs for each are different, expressing the difference in preferences that both players have over their choices.

**Figure 6:**
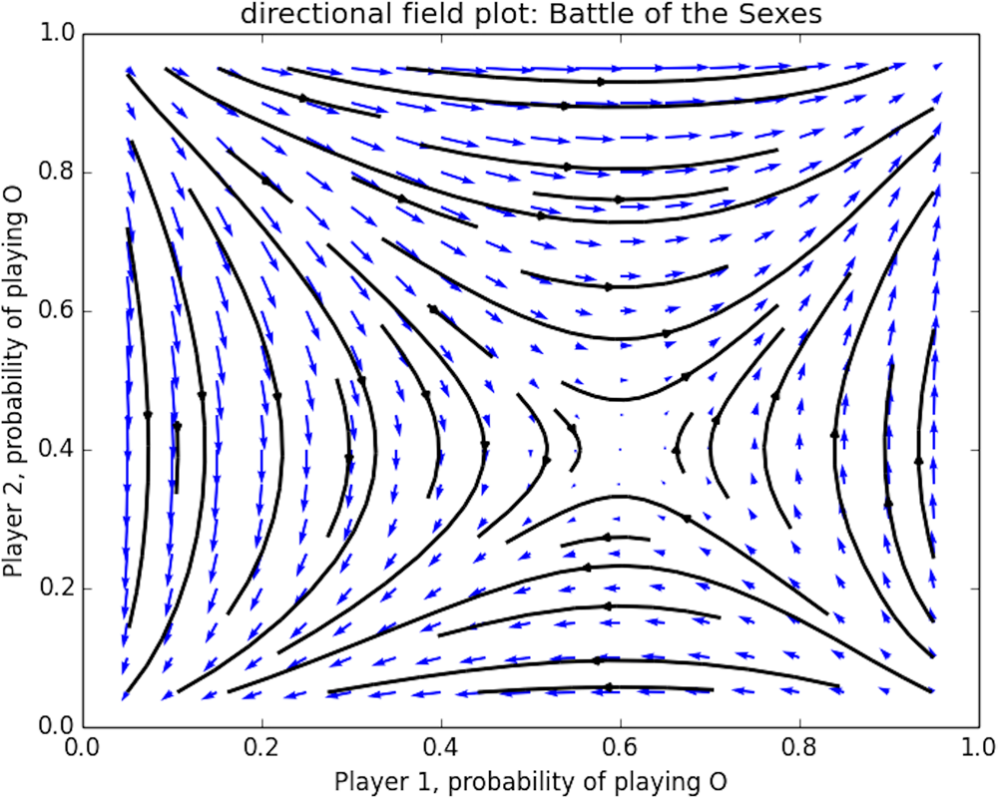
Directional field plot of the Battle of the Sexes game.

The Battle of the Sexes has two pure Nash equilibria, which are ESS as well (located at coordinates (0, 0) and (1, 1)), and one unstable completely mixed Nash equilibrium in which the players play respectively *x* = $(\frac{3}{5},\frac{2}{5})$ and *y* = $(\frac{2}{5},\frac{3}{5})$. Figure 6 illustrates the two-player evolutionary dynamics using the replicator equations, in which the x-axis corresponds to the probability with which player 1 plays *O* (Opera), and the y-axis corresponds to the probability with which the 2nd player plays *O* (Opera). The blue arrows show the vector field and the black lines are the corresponding trajectories. Note that it is still possible here to capture all of the dynamics in a static plot for the case of 2-player 2-action games, but is generally not possible in games with more than two actions.

We now use this game to illustrate Theorem 1. If we apply Theorem 1 we know that the first and second counterpart symmetric games can be described by the payoff tables shown in Table 4. The first counterpart game has $((\frac{2}{5},\frac{3}{5}),(\frac{2}{5},\frac{3}{5}))$ as a mixed Nash equilibrium, and the second counterpart game has $((\frac{3}{5},\frac{2}{5}),(\frac{3}{5},\frac{2}{5}))$ as a mixed Nash equilibrium.

**Table 4:** Counterpart matrix game 1 and 2 for the Battle of the Sexes game.

	O	M
O	3	0
M	0	2
	**O**	**M**
O	2	0
M	0	3

**Figure 7:**
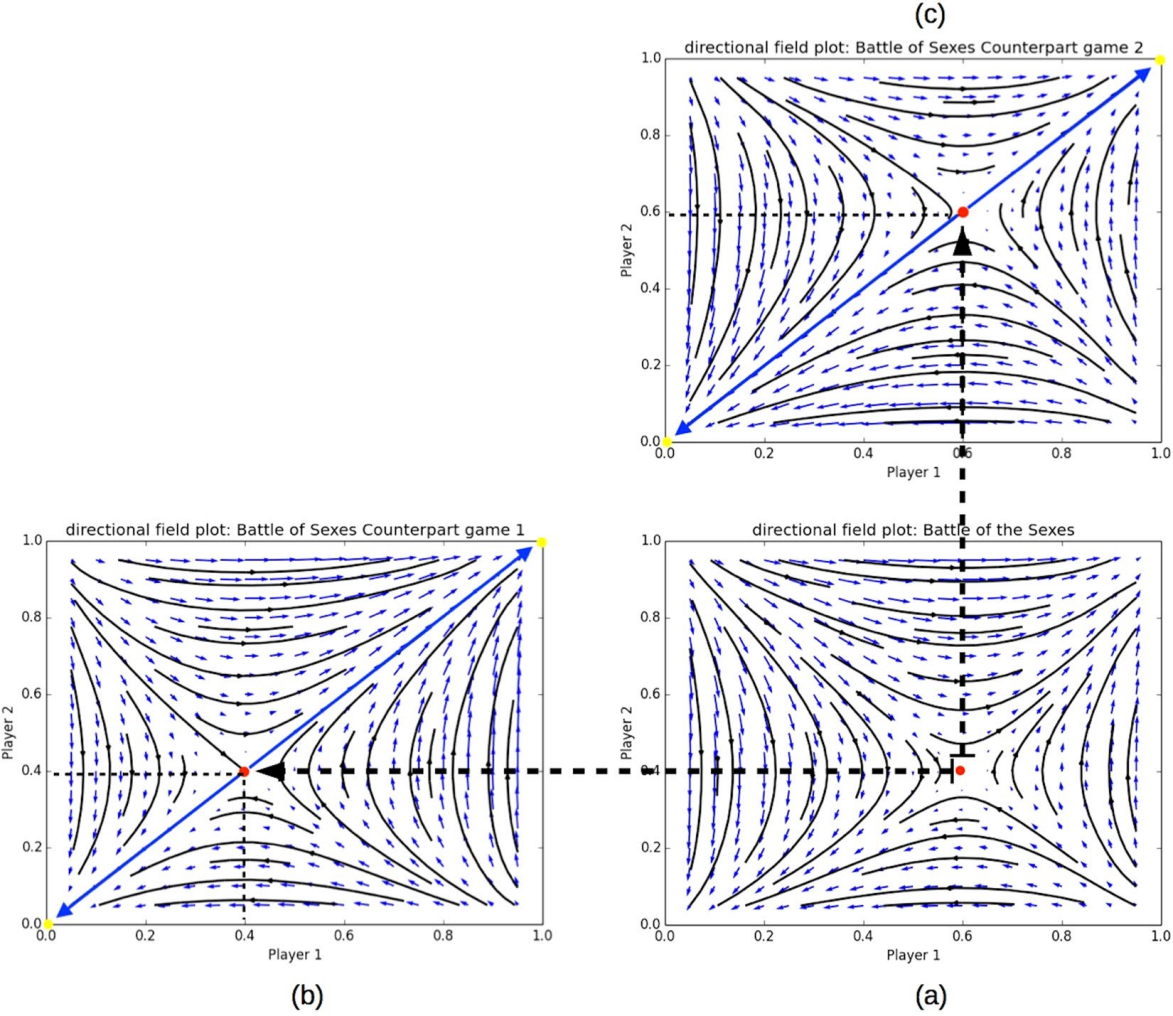
This plot shows a visual representation of how the mixed Nash equilibrium is decomposed into Nash equilibria in both counterpart games. (**a**) shows the directional field plot of the Battle of the Sexes game. (**b**) illustrates how the y-component of the asymmetric Nash equilibrium becomes a Nash equilibrium in the first counterpart game, and (**c**) shows how the x-component of the asymmetric Nash equilibrium becomes a Nash equilibrium in the first counterpart game.

In Fig. 7(b) and (c) we show the evolutionary dynamics of both counterpart games, from which the respective equilibria can be observed, as predicted by Theorem 1.

Additionally, we also know that the reverse holds, i.e., if we were given the symmetric counterpart games, we would know that $((\frac{3}{5},\frac{2}{5}),(\frac{2}{5},\frac{3}{5}))$ would also be a mixed Nash equilibrium of the original asymmetric BoS. In this case we can combine the mixed Nash equilibria of both counterpart games into the mixed Nash equilibrium of the original asymmetric game, as prescribed by Theorem 1. Specifically, as *y* = $(\frac{2}{5},\frac{3}{5})$ is part of the Nash equilibrium in the first counterpart game and *x* = $(\frac{3}{5},\frac{2}{5})$ in the second counterpart game, we can combine them into (*x* = $(\frac{3}{5},\frac{2}{5})$, *y* = $(\frac{2}{5},\frac{3}{5})$, which is a mixed Nash equilibrium of full support of the asymmetric Battle of the Sexes game.

If we now apply Theorem 2 to the Battle of the Sexes game, then we find that pure strategy Nash equilibria *x* = (1, 0) (and *y* = (1, 0) for the second counterpart) and *x* = (0, 1) (and *y* = (0, 1) for the second counterpart), which are both ESS, are also Nash equilibria in the counterpart games shown in Table 4. Also here the reverse holds, i.e., if we know the counterpart games, and we observe that *x* = (1, 0) and *x* = (0, 1) (*y* = (1, 0) and *y* = (0, 1) for the other counterpart of the game) are Nash in both games, we know that *x* = *y* = (1, 0) and *x* = *y* = (0, 1) are also Nash in the original asymmetric game. This can also be observed in Fig. 7(a),(b) and (c). Specifically, the pure Nash equilibria are situated at coordinates (0, 0) and (1, 1) in Fig. 7(b) and (c). Furthermore, it is important to understand that the counterpart dynamics are visualised only on the diagonal from coordinates (0, 0) to (1, 1), as that is where both players play with the same strategy distribution over their respective actions.

### Extended Battle of the Sexes game

In order to illustrate the theorems in a game that is non-square, including permutation of strategies, we extend the Battle of the Sexes game with a third strategy. Specifically, we give the second player a third strategy *R* in which she can choose to listen to a concert on the radio instead of going to the opera or movies with her partner. This game is illustrated in Table 5.

**Table 5:** Extended Battle of the Sexes game.

	O	R	M
O	3, 2	0.5, 0.5	0, 0
M	0, 0	0.5, 0.55	2, 3

If we would like to carry out a similar evolutionary analysis as before we need two populations for the asymmetric replicator equations. Note that in this case the strategy sets of both players are different. Using the asymmetric replicator dynamics to plot the evolutionary dynamics quickly becomes complicated since the full dynamical picture is high-dimensional and not faithfully represented by projections to the respective player’s individual strategy simplices. In other words, a static plot of the dynamics for one player does not immediately allow conclusions about equilibria, as it only describes a player’s strategy evolution assuming a fixed (rather than dynamically evolving) strategy of the other player. Again we can apply the counterpart RD theorems here to remedy this problem and consequently analyse the equilibrium structure in the symmetric counterpart games instead, yielding insight into the equilibrium landscape of the asymmetric game.

In Tables 6 and 7 we show the counterpart games A and B. Note that we introduce a *dummy* action *D* for the first player, in order to make sure that both players have the same number of actions in their strategy set (a requirement to apply the theorems) by just adding −1 for both players playing this strategy, which makes *D* completely dominated and thus redundant.

**Table 6:** Payoff matrix for the 1st counterpart game of the Extended BoS game. Strategy *D* is added to make the matrix completely square.

	O	R	M
O	3	0.5	0
M	0	0.5	2
D	−1	−1	−1

**Table 7:** Payoff matrix for the 2nd counterpart game of the Extended BoS game. Strategy *D* is added to make the matrix completely square.

	O	R	M
O	2	0.5	0
M	0	0.55	3
D	−1	−1	−1

The three Nash equilibria of interest of this asymmetric game are the following, {(*x* = (0.6, 0.4, 0),*y* = (0.4, 0, 0.6)),(*x* = (0, 1, 0),*y* = (0, 0, 1)),(*x* = (1, 0, 0),*y* = (1, 0, 0)))} (we use the online banach solver http://banach.lse.ac.uk/ to check that the Nash equilibria we find are correct^31^).

**Figure 8:**
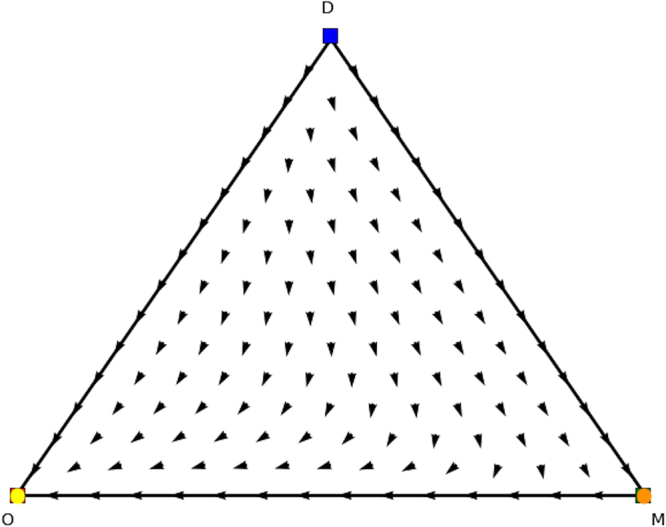
Directional field plot Σ_3_ of the first counterpart game of the extended Battle of the Sexes game.

**Figure 9:**
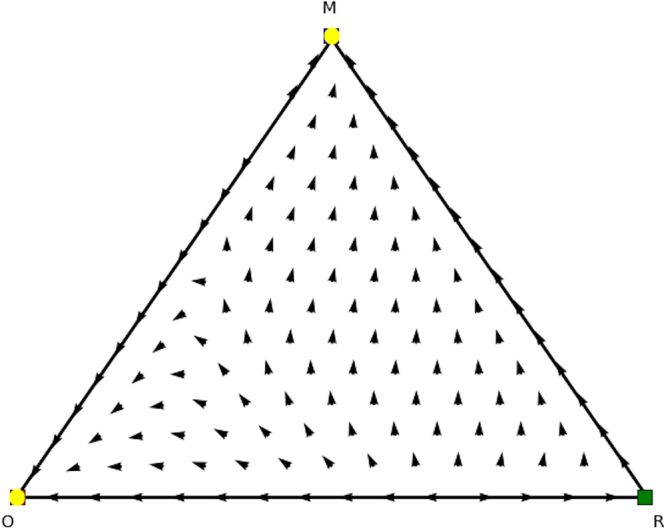
Directional field plot Σ_3_ of the second counterpart game of the extended Battle of the Sexes game.

We now look for the *y* and *x* parts of these equilibria in the counterpart games. In Fig. 8 we show the evolutionary dynamics of the first counterpart game and in Fig. 9 the evolutionary dynamics of the second counterpart game. In the first counterpart we only need to consider the 1-face formed by strategies *O* and *M* as the third strategy is our dummy strategy. In this game there are two Nash equilibria, i.e., (1, 0, 0) (stable, yellow oval) and (0, 1, 0) (unstable, orange oval), so either playing *O* or *M*. The second counterpart game also has two Nash equilibria, i.e., (1, 0, 0) and (0, 0, 1) playing either *O* or *M* as well. Note there are also two rest points at the faces formed by *O* and *R* and *O* and *M*, which are not Nash (see Fig. 5 for an explanation). There is no mixed equilibrium of full support, so we cannot apply Theorem 1 here. If we apply Theorem 2 we know that ((1, 0, 0), (1, 0, 0)) must also be a pure Nash equilibrium in the original asymmetric game, and we can remove the dummy strategy for player 1. At this stage we are left with equilibria (*x* = (0.6, 0.4, 0),*y* = (0.4, 0, 0.6)) and (*x* = (0, 1, 0),*y* = (0, 0, 1)) in the asymmetric game for which we did not find a symmetric counterpart at this stage. Now the permutation of the counterpart games, explained earlier in the findings section, comes into play. Recall that in order to study all configurations of supports of the same cardinal for both players one needs to simply permute the actions of one player in matrix *A* and *B*. Let’s have a look at such a permutation, specifically, let’s permute the 2nd and 3rd action for player 2, resulting in Tables 8 and 9.

**Table 8:** Permuted payoff matrix for the 1st counterpart game of the Extended BoS game.

	O	M	R
O	3	0	0.5
M	0	2	0.5
D	−1	−1	−1

**Table 9:** Permuted payoff matrix for the 2nd counterpart game of the Extended BoS game.

	O	M	R
O	2	0	0.5
M	0	3	0.55
D	−1	−1	−1

**Figure 10:**
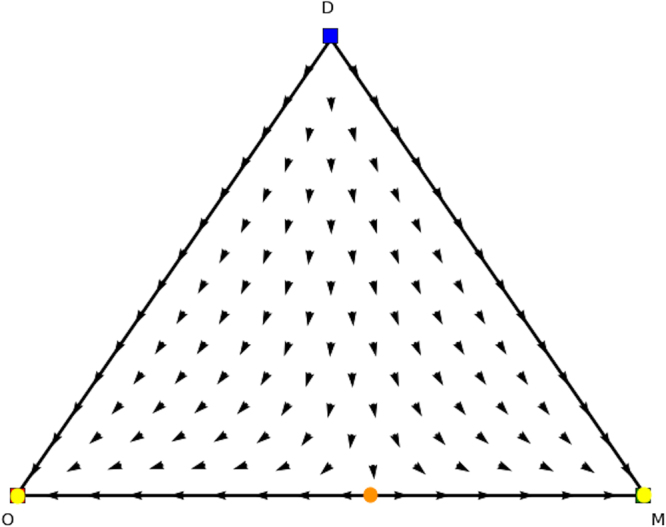
Directional field plot Σ_3_ of the first counterpart game of the permuted extended Battle of the Sexes game.

**Figure 11:**
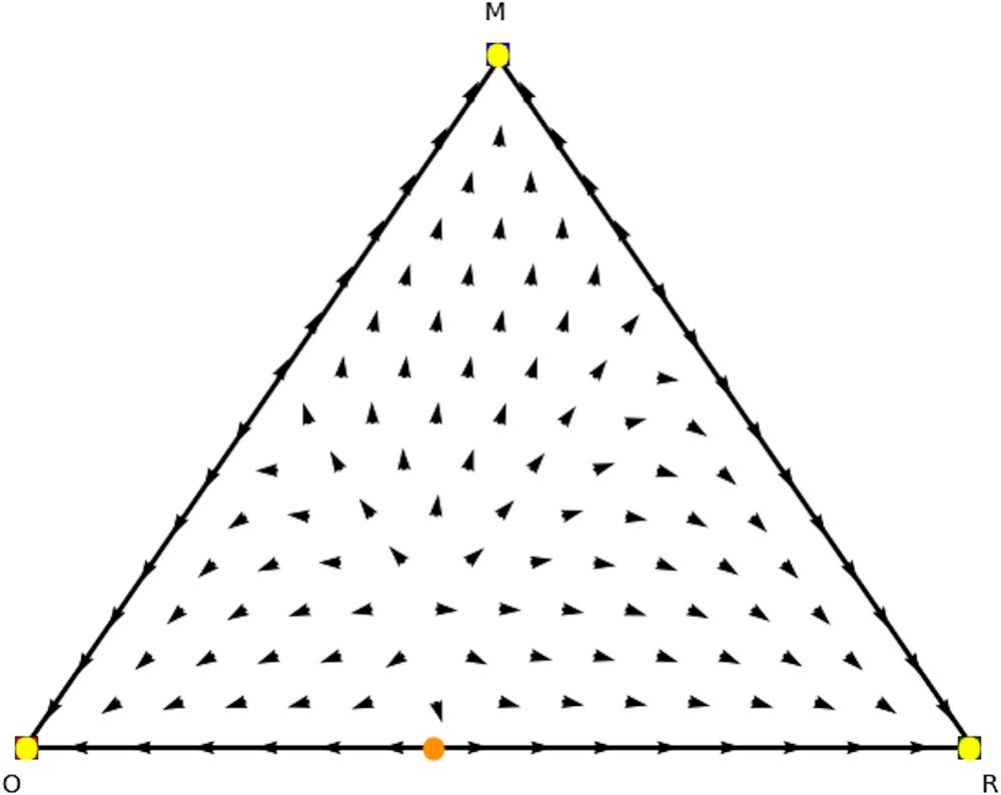
Directional field plot Σ_3_ of the second counterpart game of the permuted extended Battle of the Sexes game.

Again we can analyse these counterpart games. Specifically, we find Nash equilibria (1, 0, 0), (0.4, 0.6, 0), and (0, 1, 0) for permuted counterpart game 1 (Table 8), and Nash equilibria (0, 0, 1), (0.6, 0.4, 0), (0, 1, 0), and (1,0,0) for permuted counterpart game 2 (Table 9), which are illustrated in Figs 10 and 11. From these identified Nash equilibria in both counterpart games we can combine the remaining Nash equilibria for the asymmetric game. Specifically, by applying Theorem 2 we find (*x* = (0.6, 0.4, 0),*y* = (0.4, 0.6, 0)), which translates into (*x* = (0.6, 0.4, 0),*y* = (0.4, 0, 0.6)) for the asymmetric game as we permuted actions 2 and 3 for the second player and we need to swap these again. Additionally, we also find (*x* = (0, 1, 0),*y* = (0, 1, 0)), which translates into equilibrium (*x* = (0, 1, 0),*y* = (0, 0, 1)) for the asymmetric game as we permuted action 2 and 3 for the second player. Now we have found all Nash equilibria of the original asymmetric game.

So, also in this case, i.e., when the game is not square and strategies need to be permuted, the theorems are still applicable and allow for analysis of the original asymmetric game.

### Poker generated asymmetric games

Policy Space Response Oracles (PSRO) is a multiagent reinforcement learning process that reduces the strategy space of large extensive-form games via iterative best response computation. PSRO can be seen as a generalized form of fictitious play that produces approximate best responses, with arbitrary distributions over generated responses computed by meta-strategy solvers. PSRO was applied to a commonly-used benchmark problem in artificial intelligence research known as Leduc poker^35^. Leduc poker has a deck of 6 cards (jack, queen, king in two suits). Each player receives an initial private card, can bet a fixed amount of 2 chips in the first round, 4 chips in the second round (with a maxium of two raises in each round). Before the second round starts, a public card is revealed.

In Table 10 we present such an asymmetric 3 × 3 2-player PSRO generated game, playing Leduc Poker. In the game illustrated here, each player has three strategies that, for ease of the exposition, we call {*A*, *B*, *C*} for player 1, and {*D*, *E*, *F*} for player 2. Each one of these strategies represents a larger strategy in the full extensive-form game of Leduc poker, specifically an approximate best response to a distribution over previous opponent strategies. The game produced here then is truly asymmetric, in the sense that the strategy spaces in the original game are inherently asymmetric since player 1 always starts each round, the strategy spaces are defined by different (mostly unique) betting sequences, and even under perfect equilibrium play there is a slight advantage to player 2^9^. So, both players have significantly different strategy sets. In Tables 11 and 12 we show the two symmetric counterpart games of the empirical game produced by PSRO on Leduc poker.

**Table 10:** Payoff matrix of an asymmetric empirical game produced by PSRO applied to Leduc poker.

	D	E	F
A	−0.16, 0.16	0.05, −0.05	0.08, −0.08
B	0.64, −0.64	−0.17, 0.17	−0.31, 0.31
C	0.79, −0.79	1.06, −1.06	−0.37, 0.37

**Table 11:** First counterpart game of the Leduc poker empirical game.

	A	B	C
A	−0.16	0.05	0.08
B	0.64	−0.17	−0.31
C	0.79	1.06	−0.37

**Table 12:** Second counterpart game of the Leduc poker empirical game.

	D	E	F
D	0.16	−0.05	−0.08
E	−0.64	0.17	0.31
F	−0.79	−1.06	0.37

Again we can now analyse the landscape of equilibria of this game using the introduced theorems. Since the Leduc poker empirical game is asymmetric we need two populations for the asymmetric replicator equations. As mentioned before, analysing and plotting the evolutionary asymmetric replicator dynamics now quickly becomes very tedious as we deal with two simplices, one for each player. More precisely, if we consider a strategy for one player in its corresponding simplex, and that player is adjusting its strategy, will immediately cause the trajectory in the second simplex to change, and vice versa. Consequently, it is not straightforward anymore to analyse the dynamics and equilibrium landscape for both players, as any trajectory in one simplex causes the other simplex to change. A movie illustrates what is meant: we show how the dynamics of player 2 changes in function of player 1. We overlay the simplex of the second player with the simplex of the first player; the yellow dots indicate what the strategy of the first player is. The movie then shows how the dynamics of the second player changes when the yellow dot changes, see https://youtu.be/10m0f3iBECc.

In order to facilitate the process of analysing this game we can apply the counterpart RD theorems here to remedy the problem, and consequently analyse the game in the far simpler symmetric counterpart games that will shed light onto the equilibrium landscape of the Leduc Poker empirical game.

**Figure 12:**
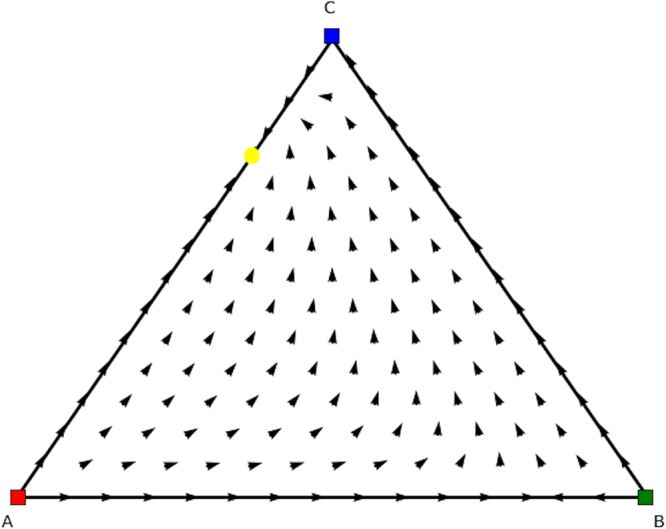
Directional field plot Σ_3_ of the first counterpart game of the Leduc poker empirical game under study.

**Figure 13:**
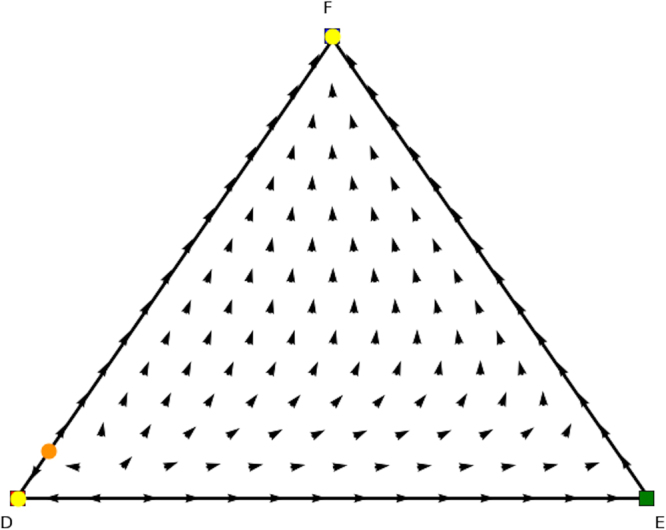
Directional field plot Σ_3_ of the second counterpart game of the Leduc poker empirical game under study.

In Figs 12 and 13 we show the evolutionary dynamics of the counterpart games. As can be observed in Fig. 12 the first counterpart game has only one equilibrium, i.e., a mixed Nash equilibrium at the face formed by *A* and *C*, which absorbs the entire strategy space. Looking at Fig. 13 we see the situation is a bit more complex in the second counterpart game, here we observe three Nash equilibria: one pure at strategy *D*, one pure at strategy *F*, and one unstable mixed equilibrium at the 1-face formed by strategies *D* and *F*. Note there is also a rest point at the face formed by strategies *D* and *E*, which is not Nash. Given that there are no mixed equilibria with full support in both games we cannot apply Theorem 1. Using Theorem 2 we now know that we only maintain the two mixed equilibria, i.e. (0.32, 0, 0.68) (CP1) and (0.83, 0, 0.17) (CP2), forming the mixed Nash equilibrium (*x* = (0.83, 0, 0.17),*y* = (0.32, 0, 0.68)) of the asymmetric Leduc poker empirical game. The other equilibria in the second counterpart game can be discarded as candidates for Nash equilibria in the Leduc poker empirical game since they also do not appear for player 1 when we permute the strategies for player 1 (not shown here).

### Mixed equilibrium of full support

As a final example to illustrate the introduced theory, we examine an asymmetric game, that has one completely mixed equilibrium and several equilibria in its counterpart games. The bimatrix game (*A*,*B*) is illustrated in Table 13 and its symmetric counterparts are shown in Tables 14 and 15.

**Table 13:** Payoff matrix of an asymmetric game with mixed equilibrium of full support.

	D	E	F
A	1, 0	0, 1	2, 0
B	0, 1	2, 0	0, 0
C	2, 0	0, 0	1, 1

**Table 14:** First counterpart game of the asymmetric game.

	A	B	C
A	1	0	2
B	0	2	0
C	2	0	1

**Table 15:** Second counterpart game of the asymmetric game.

	D	E	F
D	0	1	0
E	1	0	0
F	0	0	1

The asymmetric game has a unique completely mixed Nash equilibrium with different mixtures for the two players, i.e., (*x* = $(x=(\frac{1}{3},\frac{1}{3},\frac{1}{3});\,y=(\frac{2}{7},\frac{3}{7},\frac{2}{7}))$.

The two symmetric counterpart game each have seven equilibria. Counterpart game 1 (Table 14), has the following set of Nash equilibria: {(*a*)(*p*_1_ = $({p}_{1}=(\frac{2}{7},\frac{3}{7},\frac{2}{7}),\,{p}_{2}=(\frac{2}{7},\frac{3}{7},\frac{2}{7}))$, $({p}_{1}=(\frac{1}{2},\frac{1}{2},0),\,{p}_{2}=(0,\frac{1}{2},\frac{1}{2}))$, (*p*_1_ = (1,0,0), *p*_2_ = (0,0,1)), (*b*)(*p*_1_ = (0,1,0), *p*_2_ = (0,1,0)), ((*c*) *p*_1_ = $((c)\,{p}_{1}=(\frac{1}{2},0,\frac{1}{2}),\,{p}_{2}=(\frac{1}{2},0,\frac{1}{2}))$, (*p*_1_ = (0,0,1), *p*_2_ = (1,0,0)), (*p*_1_ = $({p}_{1}=(0,\frac{1}{2},\frac{1}{2}),\,{p}_{2}=(\frac{1}{2},\frac{1}{2},0))$}. Note that there are also two rest points, which are not Nash, at the faces formed by *A* and *B* and *B* and *C*. From these seven equilibria only (a), (b) and (c) are of interest since these are symmetric equilibria in which both players play with the same strategy (or support). Also counterpart game 2 has seven equilibria, i.e., $\{(d)\,({p}_{1}=(\tfrac{1}{3},\tfrac{1}{3},\tfrac{1}{3}),{p}_{2}=(\tfrac{1}{3},\tfrac{1}{3},\tfrac{1}{3}))$, $({p}_{1}=(\tfrac{1}{2},0,\tfrac{1}{2}),{p}_{2}=(0,\tfrac{1}{2},\tfrac{1}{2}))$, $((e){p}_{1}=(0,0,1),{p}_{2}=(0,0,1))$, $({p}_{1}=(0,\tfrac{1}{2},\tfrac{1}{2}),{p}_{2}=(\tfrac{1}{2},0,\tfrac{1}{2}))$, $((f){p}_{1}=(\tfrac{1}{2},\tfrac{1}{2},0),{p}_{2}=(\tfrac{1}{2},\tfrac{1}{2}),0)$, $({p}_{1}=(1,0,0),{p}_{2}=(0,1,0))$, $({p}_{1}=(0,1,0),{p}_{2}=(1,0,0)\}$ of which only (d), (e) and (f) are of interest.

**Figure 14:**
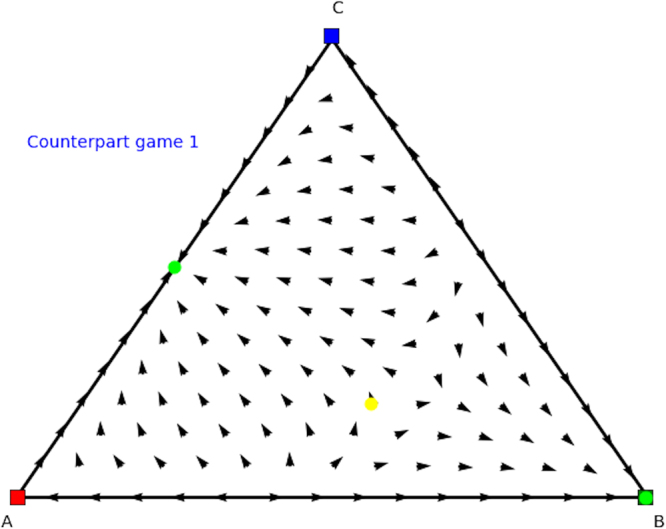
Directional field plot Σ_3_ of the first counterpart game of the mixed equilibrium asymmetric game.

**Figure 15:**
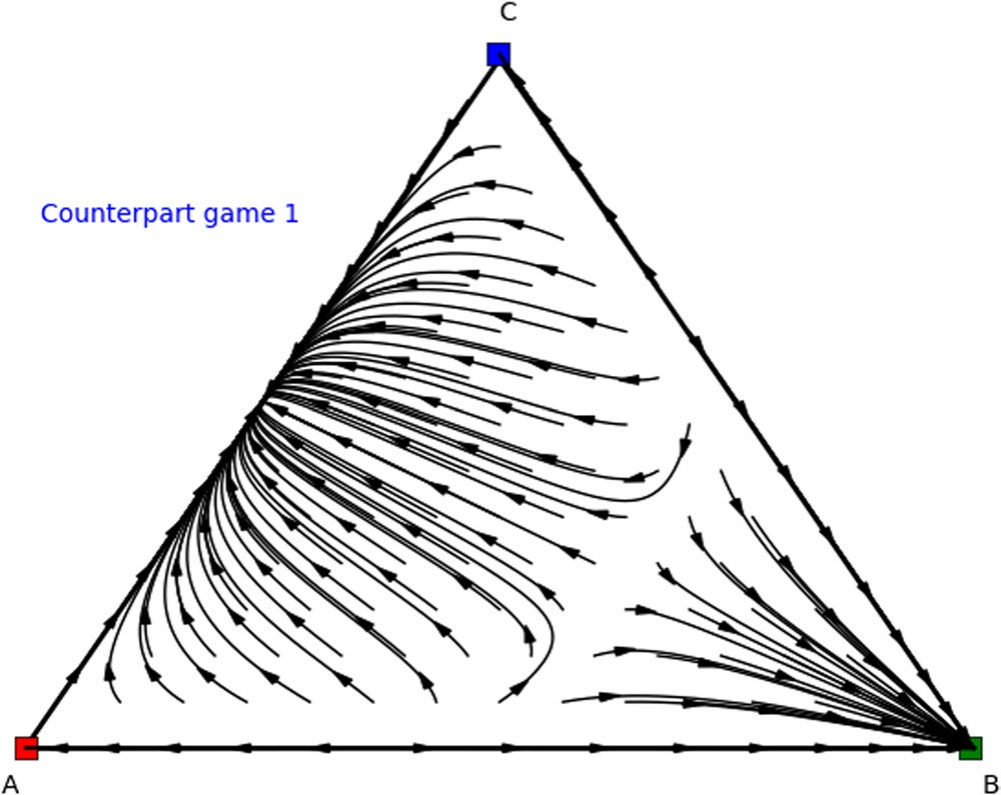
Trajectory plot Σ_3_ of the first counterpart game of the mixed equilibrium asymmetric game.

**Figure 16:**
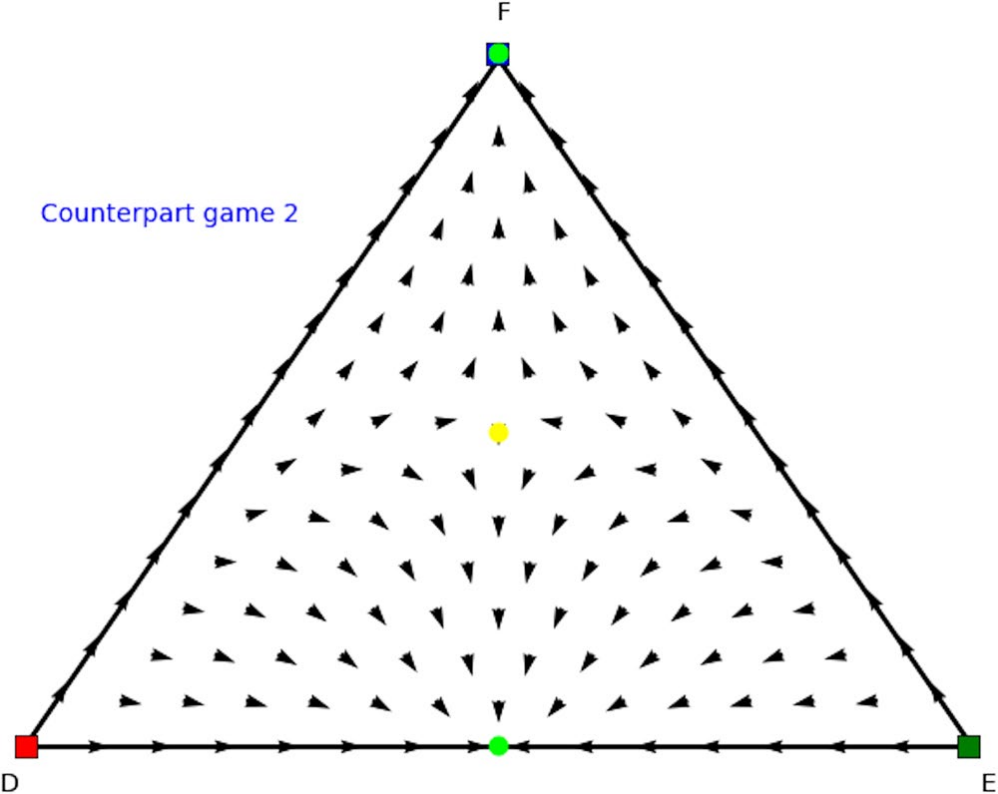
Directional field plot Σ_3_ of the second counterpart game of the mixed equilibrium asymmetric game.

**Figure 17:**
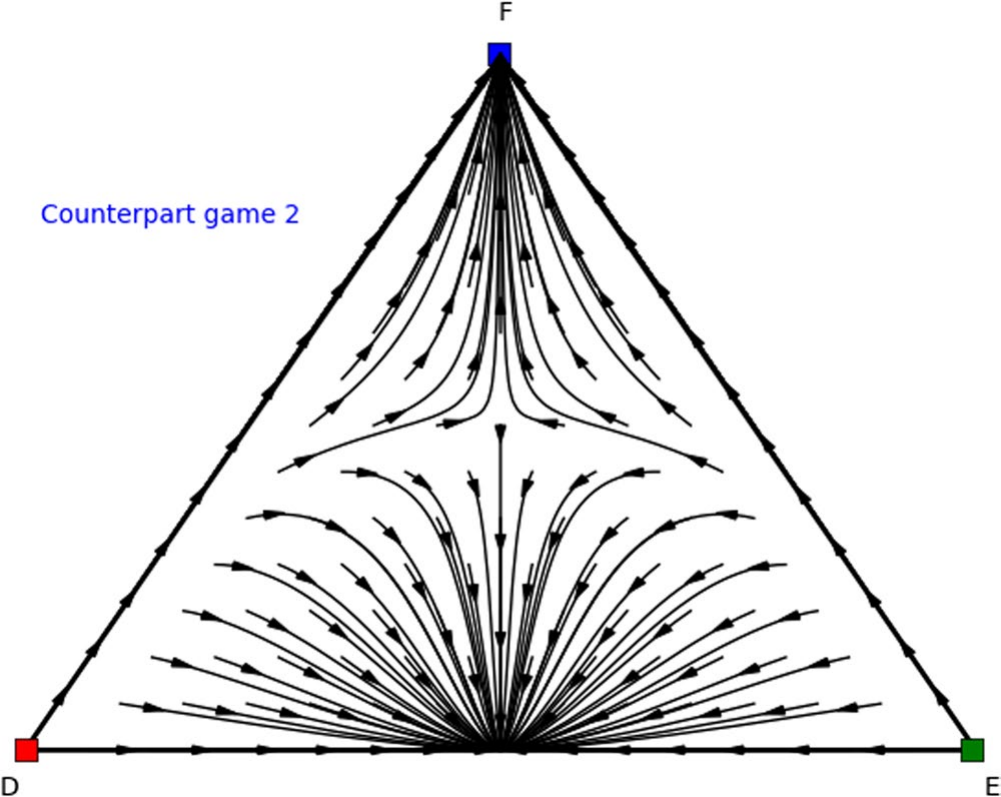
Trajectory plot Σ_3_ of the second counterpart game of the mixed equilibrium asymmetric game.

We observe that only the completely mixed equilibrium of the asymmetric game, i.e., $(x=(\frac{1}{3},\frac{1}{3},\frac{1}{3});\,y=(\frac{2}{7},\frac{3}{7},\frac{2}{7}))$, has its counterpart in the symmetric games. To apply the theorems we only need to have a look at equilibria (a), (b) and (c) in counterpart game 1, and (d), (e) and (f) in counterpart game 2. These equilibria can also be observed in the directional field plots, respectively, trajectory plots, illustrating the evolutionary dynamics of both counterpart games in Figs 14, 15, 16 and 17. Figure 14 visualises the three remaining equilibria (a), (b) and (c), with (a) indicated as a yellow oval, and (b) and (c) both indicated as green ovals. As can be observed, (a) is an unstable mixed equilibrium, (b) is a stable pure equilibrium, and (c) is a partly mixed equilibrium at the 2-face formed by strategies A and C.

We can make the same observation for the second counterpart game, and see that (d), (e) and (f) are equilibria in Fig. 16. Equilibrium (d), indicated by a yellow oval, is completely mixed, equilibrium (e) is a pure equilibrium in corner F (green oval), and (f) is a partly mixed equilibrium on the 2-face formed by strategies D and E (green ovals as well).

If we now apply Theorem 1 we know that we can combine the mixed equilibria of full support of both counterpart games into the mixed equilibrium of the original asymmetric game, in which the mixed equilibrium of counterpart game 1, i.e. $(\frac{2}{7},\frac{3}{7},\frac{2}{7})$, becomes the part of the mixed equilibrium in the asymmetric game of player 2, and the mixed equilibrium of counterpart game 2, i.e. $(\frac{1}{3},\frac{1}{3},\frac{1}{3})$, becomes the part of the mixed equilibrium in the asymmetric game of player 1, leading to $(x=(\frac{1}{3},\frac{1}{3},\frac{1}{3});\,y=(\frac{2}{7},\frac{3}{7},\frac{2}{7}))$. Both equilibria are unstable in the counterpart games and also form an unstable mixed equilibrium in the asymmetric game.

## Discussion

Replicator Dynamics have proved to be an excellent tool to analyse the Nash landscape of multiagent interactions and distributed learning in both abstract games and complex systems^1,2,4,6^. The predominant approach has been the use of symmetric replicator equations, allowing for a relatively straightforward analysis in symmetric games. Many interesting real-world settings though involve roles or player-types for the different agents that take part in an interaction, and as such are *asymmetric* in nature. So far, most research has avoided to carry out RD analysis in this type of interactions, by either constructing a new symmetric game, in which the various actions of the different roles are joined together in one population^23,24^, or by considering the various roles and strategies as heuristics, grouped in one population as well^2,3,8^. In the latter approach the payoffs due to different player-types are averaged over many samples of the player type resulting in a single average payoff to each player for each entry in the payoff table.

The work presented in this paper takes a different stance by decomposing an asymmetric game into its symmetric counterparts. This method proves to be mathematically simple and elegant, and allows for a straightforward analysis of asymmetric games, without the need for turning the strategy spaces into one simplex or population, but instead allows to keep separate simplices for the involved populations of strategies. Furthermore, the counterpart games allow to get insight in the type and form of interaction of the asymmetric game under study, identifying its equilibrium structure and as such enabling analysis of abstract and empirical games discovered through multiagent learning processes (e.g. Leduc poker empirical game), as was shown in the experimental section.

A deeper counter-intuitive understanding of the theoretical results of this paper is that when identifying Nash equilibria in the counterpart games with *matching* support (including permutations of strategies for one of the players), it turns out that also the combination of those equilibria form a Nash equilibrium in the corresponding asymmetric game. In general, the vector field for the evolutionary dynamics of one player is a function of the other player’s strategy, and hence a vector field in one player’s simplex doesn’t carry much information as any equilibria you observe in it are changing with time as the other player is moving too. However, if you position the second player at a Nash equilibrium, it turns out that player one becomes indifferent between his different strategies, and remains stationary under the RD. This gives the unique situation in which the vector field plot for the second player’s simplex is actually meaningful, because the assumption of player one being stationary actually holds (and vice versa). This is what we end up using when establishing the correspondence of the Nash Equilibria in asymmetric and counterpart games, and why the single-simplex plots for the counterpart games are actually meaningful for the asymmetric game - but this is also why they only describe the Nash Equilibria faithfully, but fail to be a valid decomposition of the full asymmetric game away from equilibrium.

These findings shed new light on asymmetric interactions between multiple agents and provide new insights that facilitate a thorough and convenient analysis of asymmetric games. As pointed out by Veller and Hayward^36^, many real-world situations, in which one aims to study evolutionary or learning dynamics of several interacting agents, are better modelled by asymmetric games. As such these theoretical findings can facilitate deeper analysis of equilibrium structures in evolutionary asymmetric games relevant to various topics including economic theory, evolutionary biology, empirical game theory, the evolution of cooperation, evolutionary language games and artificial intelligence^11,12,37–40^.

Finally, the results of this paper also nicely underpin what is said in H. Gintis’ book on the evolutionary dynamics of asymmetric games, i.e., *‘although the static game pits the row player against the column player*, *the evolutionary dynamic pits row players against themselves and column players against themselves’*^32^ (chapter 12, p.292). He also indicates that this aspect of an evolutionary dynamic is often misunderstood. The use of our counterpart dynamics supports and illustrates this statement very clearly, showing that in the counterpart games species play games within a population and as such show an intra-species survival of the fittest, which is then combined into an equilibrium of the asymmetric game.
